# A binary biclustering algorithm based on the adjacency difference matrix for gene expression data analysis

**DOI:** 10.1186/s12859-022-04842-4

**Published:** 2022-09-19

**Authors:** He-Ming Chu, Jin-Xing Liu, Ke Zhang, Chun-Hou Zheng, Juan Wang, Xiang-Zhen Kong

**Affiliations:** 1 School of Computer Science, Qufu Normal University, Rizhao, 276826 China; 2 Department of Oncology, Rizhao People’s Hospital, Rizhao, 276826 China

**Keywords:** Biclustering, Gene expression data, Adjacency matrix, Binary data

## Abstract

Biclustering algorithm is an effective tool for processing gene expression datasets. There are two kinds of data matrices, binary data and non-binary data, which are processed by biclustering method. A binary matrix is usually converted from pre-processed gene expression data, which can effectively reduce the interference from noise and abnormal data, and is then processed using a biclustering algorithm. However, biclustering algorithms of dealing with binary data have a poor balance between running time and performance. In this paper, we propose a new biclustering algorithm called the Adjacency Difference Matrix Binary Biclustering algorithm (AMBB) for dealing with binary data to address the drawback. The AMBB algorithm constructs the adjacency matrix based on the adjacency difference values, and the submatrix obtained by continuously updating the adjacency difference matrix is called a bicluster. The adjacency matrix allows for clustering of gene that undergo similar reactions under different conditions into clusters, which is important for subsequent genes analysis. Meanwhile, experiments on synthetic and real datasets visually demonstrate that the AMBB algorithm has high practicability.

## Introduction

In recent years, the binary data matrix has been used in a variety of fields, including bioinformatics [1, 2], data mining [3–5], data analysis [6], etc. A binary dataset is a data matrix about the relationship between a set of objects[7]. Only two elements in a binary matrix are 0 and 1. For the biclustering algorithm that processes the binary data matrix converted from gene expression data, we call it binary biclustering algorithm for convenience. The biclusters obtained by binary biclustering algorithms are considered to be submatrices with all 1. And a bicluster is considered statistically significant when the number of 1’s in it is large enough [8]. Noteworthy, the meaning of 0 and 1 in the binary matrix could be known by combining the context. In this paper, the values of 1 and 0 indicate, respectively, whether the gene reacts under certain conditions. So far, a host of binary biclustering algorithms have been investigated by researchers. For example, the Bimax algorithm, proposed by Prelić et al*.* [9], is a divide-and-conquer algorithm that yields a maximal binary submatrix in which important information represents 1 or 0. The BiBit algorithm proposed by Domingo et al*.* [7] is to obtain biclusters by row coding. In binary matrices, the element values have the same meaning as Bimax. It uses the parameters $\min_{r}$ and $\min_{c}$ to obtain the final bicluster, and both $\min_{r}$ and $\min_{c}$ are equal to 2. Furthermore, the BiBinAlter algorithm proposed by Saber et al*.* [10], the Binary Matrix Factorization (BMF) proposed by Zhang et al*.* [11], the QUBIC2 algorithm proposed by Xie et al*.* [12] are excellent binary biclustering algorithms. In particular, the QUBIC2 algorithm use various preprocessing methods to convert gene expression data into a binary data matrix and obtain biclustering. Noteworthy, columns with value of 1 in binary matrix represents the same properties.

Biclustering methods are proved to be NP-hard problem [13]. Therefore, according to the solution of the algorithm, the biclustering algorithms can be classified into various types [14, 15]. Nevertheless, two major categories can be classified from the data, binary biclustering and non-binary biclustering algorithms. Comparing the binary biclustering algorithm with the non-binary biclustering algorithm, it can be found that two kinds of biclustering algorithm deal with different datasets. The non-binary biclustering algorithms can process gene expression data directly, such as Local Search Algorithms (LSM) [16], while binary biclustering algorithms convert gene expression data into a binary data matrix before processing the expression dataset. For the binary biclustering algorithm, one of the termination conditions of the Bimax algorithm is that the resulting submatrix no longer contains 0 elements. In addition, the BiBit algorithm eventually obtains biclusters by selecting columns with a value of 1 in the encoding. The non-binary biclustering algorithm will obtain the biclusters directly according to its algorithmic steps. Cheng and Church algorithm (CC) is the first biclustering algorithm to be applied to gene expression data [17]. It adds or removes data from the seed according to the Mean Squared Residue (MSR) function. When the MSR value of an added element is greater than the threshold value, it means that the element cannot be added to the seed set. The Scaling Mean Squared Residue (SMSR) method similar to the CC algorithm is proposed by Mukhopadhyay et al. [18]. The algorithm is also based on MSR values to obtain biclusters. Direct clustering (DC) proposed by Hartigan et al*.* [19] is among the first published biclustering algorithms applied to data matrices. The Flexible Overlapped biclustering (FLOC) algorithm proposed by Yang et al*.* [20] is a stochastic iterative greedy algorithm. It initializes k biclustered and adds rows and columns to the biclusters according to the given probability.

The binary biclustering algorithm belongs to the special biclustering algorithm, which mainly processes the binary matrix to obtain the optimal biclusters. Nevertheless, to obtain the co-expression cluster, binary biclustering algorithms fail to make the suitable balance between performance and running time. For example, in large expression data, Bimax algorithm has fast speed but lost better performance, while the BiBit algorithm has outperformance but slow running speed. To balance between running time and performance, in this paper, a new binary biclustering algorithm based on constructing adjacency difference matrices is proposed, called the Adjacency Difference Matrix Binary Biclustering (AMBB) algorithm. In addition, the AMBB algorithm does not require to encode and traverse all rows for continuous seed acquisition. And based on the adjacency difference matrix, the AMBB algorithm solves the problem of clustering genes with similar responses under certain conditions. Moreover, the performance of the AMBB algorithm is tested using synthetic and real datasets. The experimental results show that the AMBB algorithm outperforms BiBit, QUBIC and Bimax algorithms in the synthetic dataset, and the AMBB algorithm can obtain a large number of valid genes in real dataset, which is very important for further analysis of gene expression data.

## Methods

The AMBB algorithm uses the row with the highest number of 1’s in the binary matrix as the seed, and iterates the row and column elements continuously according to the adjacency difference matrix to obtain a bicluster. Significantly, the adjacency difference matrix is constructed based on the seed.

The parameters for the AMBB algorithm are row adjacency difference matrix threshold $\delta$ and column adjacency difference matrix threshold $\lambda$, in which $\delta$ is used to control the selection of rows and $\lambda$ is used to control the selection of columns.

### Declaration

An input pre-processed binary data matrix is defined as $E = (I,J)$, where $I$ and $J$ are two finite sets, denoting the set of rows and the set of columns, respectively. For gene expression data, we define rows to represent genes and columns to represent conditions. Furthermore, when the gene $i \in I$ reacts under condition $j \in J$, element $x_{ij}$ in the binary matrix has a value of 1, otherwise it is 0.

The binary data matrix $E = (I,J)$, with $n = \left| I \right|$ and $m = \left| J \right|$, can be constructed as a $n * n$ row difference matrix and a $m * m$ column difference matrix. The element of the row performs $\oplus (OR)$ operation on the seed element, and these values obtained are summed up. The result is called the row difference value $\eta_{ii^{\prime}}$. Where $i^{\prime}$ represents the $i$-th row. The column difference value $\eta_{jj^{\prime}}$ is calculated in the same way. And as same as the row difference value, $j^{\prime}$ represents the $j$-th column.

In the row difference value matrix, each row represents the vector of value differences between the seed and all rows in the binary matrix. Similarly, the rows and columns of the column difference value matrix are expressed in terms of the same meaning as the row difference value matrix. For an instance, there is a binary matrix E = (1, 1, 0, 0; 1, 1, 1, 0). The row difference matrix is r = (0, 1; 1, 0). Therefore, two rows in E are be selected and the column difference matrix is c = (0, 0, 1, 2; 0, 0, 1, 2; 1, 1, 0, 1; 2, 2, 1, 0).

The submatrix $E^{\prime}$ is called the maximum bicluster if there are no elements with value 0 in this submatrix. For above case, E’ = (1, 1; 1, 1).

### Parameter

We argue that a large number of biclusters can effectively identify biclusters of different patterns. Therefore, we take the number of biclusters as the basis for the determination of parameters. In the AMBB algorithm, there are two parameters, that is, row difference threshold $$\delta$$ and the column difference threshold $$\lambda$$. The row difference value matrix was constructed based on $$i$$-th ($$1 \le i \le n$$) row. When the $$\eta_{ii^{\prime}}$$ $$(i < i^{\prime} \le n)$$ is smaller than the row threshold $$\delta$$, $$i$$ th row as seed and $$i^{\prime}$$ row is added into it. Then the column difference value matrix was constructed based on $$j$$ th ($$1 \le j \le m$$) column. When $$\eta_{jj^{\prime}}$$$$(j < j^{\prime} \le m)$$ is smaller than the column threshold $$\lambda$$, $$j$$ th column is can be seen bicluster and $$j^{\prime}$$ is added into it. Therefore, it is very important to choose a suitable threshold value. When an algorithm is able to get the best threshold automatically, then it will be easier to get the best cluster for the operation of this algorithm.

To select the optimal threshold, we propose a new method for semi-automatic selection of threshold values. This is binary search. For example, one dataset contains 10,000 genes. And according to the adjacency difference matrix, the difference value range is [500, 1000]. First, the threshold $\delta$ is set to 750 $\pm$ 50 (1%*r) where r represents the number of the currently gross genes. And then set $\delta$ are 625 $\pm$ 12 (1%*r) and 875 $\pm$ 12(1%*r). Assuming that in this dataset, the parameter $\delta$ selection range is 750 $\pm$ 50(1%*r). It means that if the value of $\delta$ is smaller than this range, the number of co-expression clusters is relatively less, and on the contrary, the number of clusters does not change much. At this time, the selection range of parameters is changed from (500,1000) to (625,875). Within the new range, the binary search continues until the final threshold value is determined. This approach solves the problem of selecting the optimal threshold for the AMBB algorithm.

**Fig. 1:**
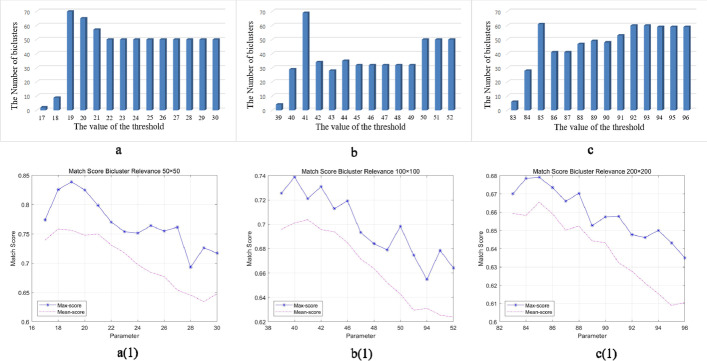
A schematic of the biclustered clusters obtained for each row threshold. **a** The number of biclusters result of the synthetic dataset of 50*50. **b** The number of biclusters result of 100*100 synthetic dataset. **c** The number of biclusters result of 200*200 synthetic dataset

Considering that the sizes of binary data matrix are not the same, the density of 1 is also different. In a synthetic binary data matrix, the 1’density represents noise level. A threshold range is given to AMBB, and the algorithm is run once for each threshold, keeping the optimal bicluster obtained by this algorithm. When the AMBB algorithm runs all the thresholds, the one of them best is selected according to the number of biclusters. The threshold that obtained the largest number of biclusters is the optimal. To get the similar columns, the threshold of columns is initially set to $\left\lceil {r/2} \right\rceil$ where $r$ represents the number of rows in current set. Each iteration of the column threshold in the same submatrix starts from 0 and automatically incremented one until the threshold value is equal to $\left\lceil {r/2} \right\rceil$ or a bicluster is obtained. The selection of row thresholds is illustrated in Fig. 1. Noteworthily, the 1’s density in the synthetic dataset is set at 50%. And the implanted biclusters are shift pattern and the number is 10. Three different sizes of synthetic data were used to select the optimal threshold and each dataset was run 100 times. The threshold that generates the largest number of biclusters is most likely to be the optimal row threshold, and use the threshold as a benchmark to find the optimal threshold.

In Fig. 1, the ordinate a to c and a (1) to c (1) represent the number of biclusters and the Relevance value, respectively. Horizontal axes represents the row threshold for six graphs. For the Relevance score, it has detailed description in “Evaluation Metric” of section “RESULTS”. a (1) and a show the result of 50*50 synthetic dataset and the optimal row threshold is 19. b (1) and b show the result of 100*100 synthetic dataset and the optimal row threshold is 40. In 200*200 synthetic dataset, c (1) shows the optimal row threshold is 85.

Noteworthily, running ten times for each dataset, the Max-score represents maximum score and the Mean-score represents mean score. To summarize the selection of row thresholds, we simply enter a threshold range within which the AMBB algorithm finds the threshold that yields the maximum number of biclusters, and then uses this threshold as a benchmark to find the optimal parameter for the current data.

### Algorithm

The first step of the AMBB algorithm converts the gene expression data into a binary matrix. Data conversion using the preprocessing method proposed by the Bimax algorithm. A threshold is calculated based on the expression value of the gene in all samples. The calculation formula is $x_{\min } + (x_{\max } + x_{\min } )/2$. Where $x_{\min }$ and $x_{\max }$ represent the minimum and maximum expression value in data matrix respectively. If $x_{ij}$, the expression value of the $i$ th row and $j$ th column, is greater than the current threshold, the corresponding position value in the binary matrix is 1, otherwise 0.

**Fig. 2:**
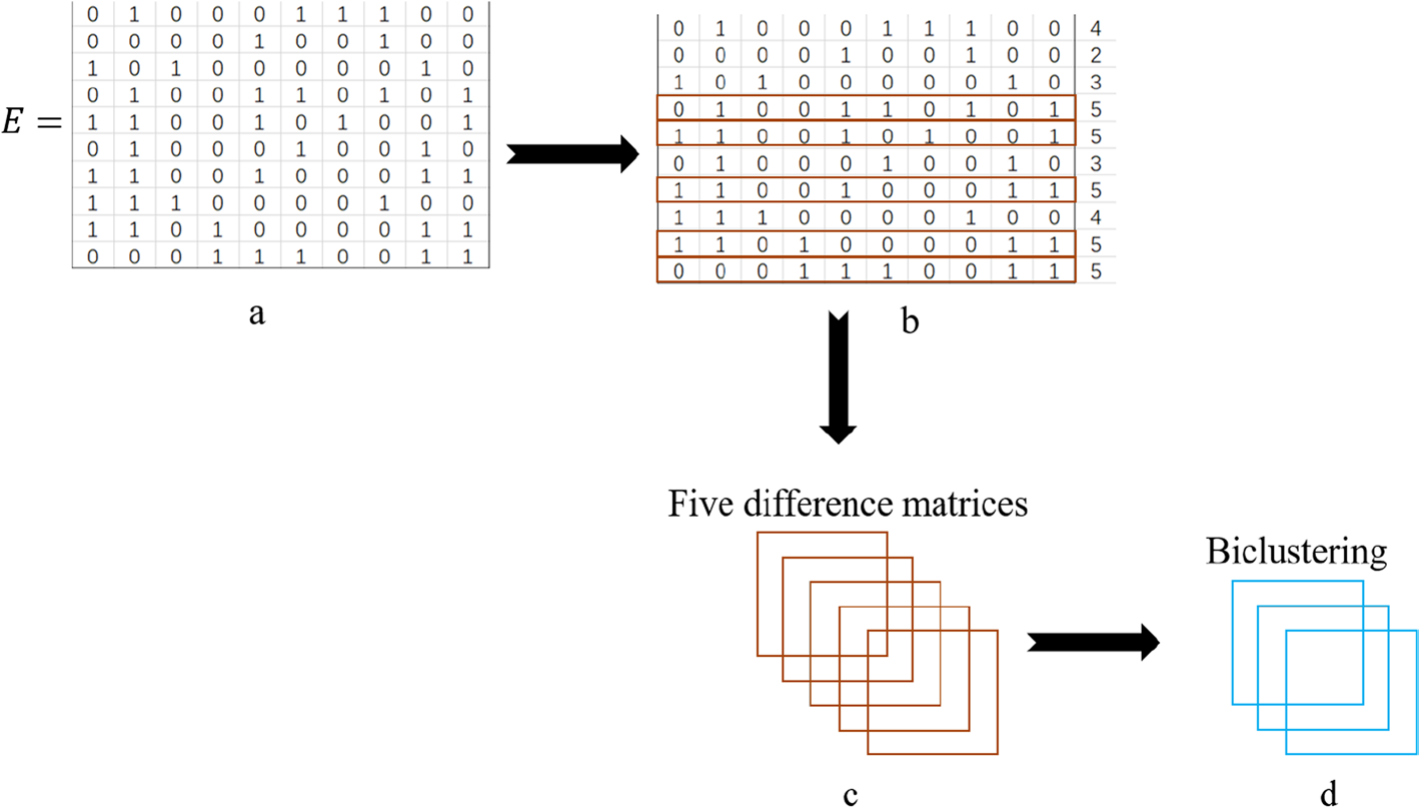
A brief schematic of the AMBB algorithm. **a** shows the pre-processing matrix. **b** shows the selection of seeds. **c** shows the construction of the difference matrix, and (**d**) shows the acquisition of biclusters

**Fig. 3:**
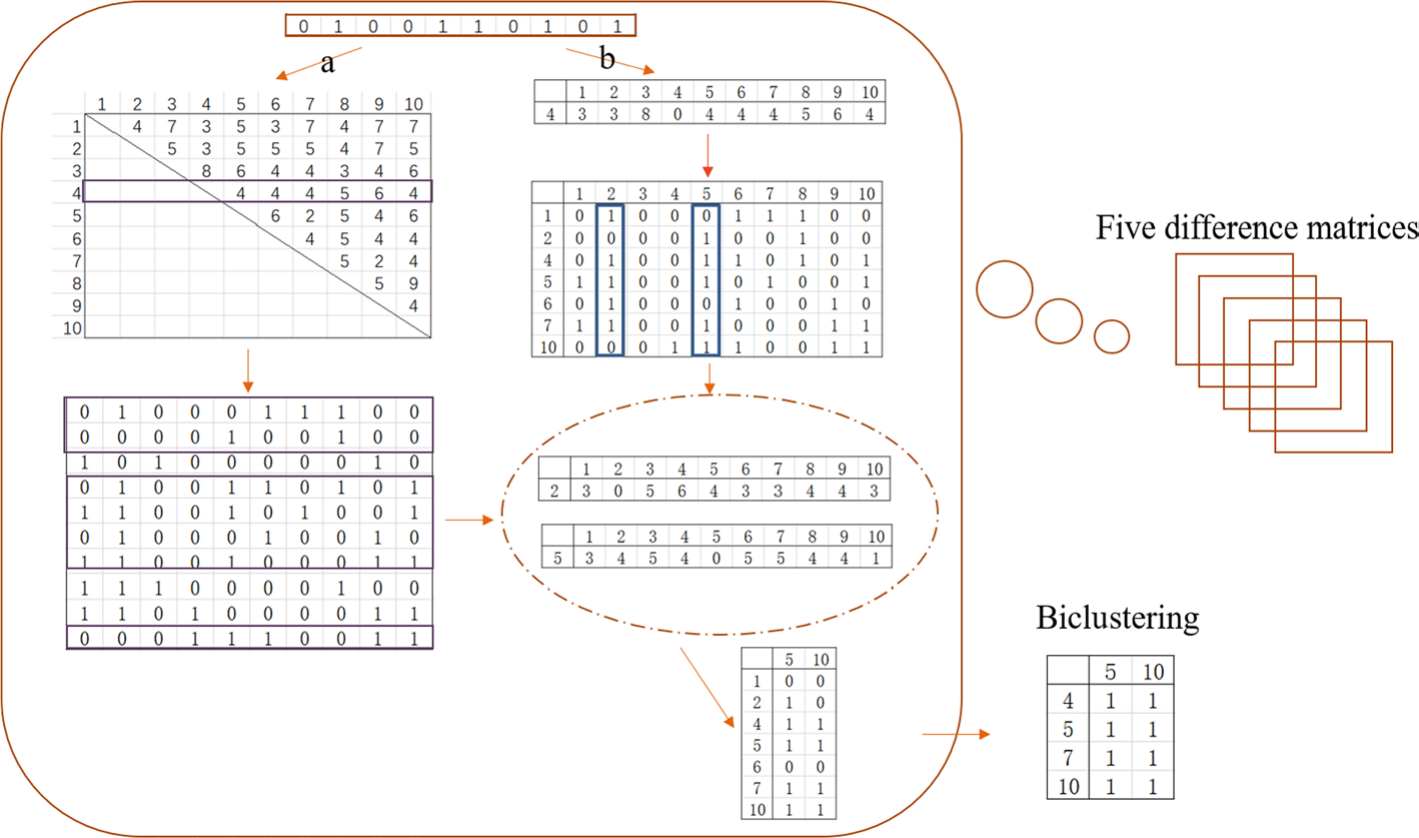
The diagram of two methods for two obtained seed methods of AMBB. Methods **a** and method **b** show two methods of obtaining seeds

The schematic diagram of the algorithm is shown in Fig. 2. The main steps of the algorithm are to select the seed and construct the adjacency difference matrix. There are two ways to obtain seeds. The first way is to use each row as a seed to construct a row difference matrix. The other is to use the row with the highest number of 1’s in it as a seed first. Figure 3 shows two methods in detail. To find the optimal method, we compared the two approaches and present detailed results are presented in the next section.

Figure 2a represents the input binary matrix. Figure 2b shows the selection of seeds, which is simply the selection of the row with the highest number of 1 from the binary matrix. Five rectangles exist in Fig. 2c contains five rectangles representing five difference matrices from five seeds, each containing a row difference matrix and a column difference matrix. Figure 2d shows three biclusters. Therefore, a seed may not have biclusters according to the threshold value.

Once the seeds are selected, the AMBB algorithm then constructs a row difference matrix and a column difference matrix. Figure 3 depicts two obtained seed methods obtained from AMBB algorithm, denoted as method $a$ (a-AMBB) and method $b$ (b-AMBB). Method $b$ selects the row with the largest row value as the seed, and then constructs a row difference matrix based on that row, where the matrix dimension is $1*m$. The difference value (DV) is defined as follows:

1 $$DV_{si} = \sum\limits_{j = 1}^{m} {(x_{ij} \oplus x_{sj} )} ,$$

where $DV_{si}$ denotes the difference value between seed $s$ and row $i$. $x_{ij}$ denotes the $j$-th column of the $i$-th row, and $x_{sj}$ denotes the $j$-th column of the seed.

If the $DV$ value is less than the threshold $\delta$, the $i$-th row is put into the row cluster. When all the rows have been computed, a cluster of rows expanded by seeds is then obtained. The seed in Fig. 3 is the fourth row of the binary matrix, and the difference value matrix is $1*10$. Note that in this example, we set a row threshold $\delta$ of 5. Put rows with difference value less than 5 together to form a row cluster. The column values are calculated for each column in the row cluster, and the column values are calculated as follows:

2 $$CV_{j} = \sum\limits_{i \in I^{\prime},j \in J} {x_{ij} } ,$$

where $CV_{j}$ denotes the column value of the $j$-th column. $I^{\prime}$ indicates row cluster. The column with the largest column value in $I^{\prime}$ is given priority as the column seed, and the column difference matrix is constructed. Normally the column threshold $\lambda$ is set to 1 for better acquisition of biclusters. In the example of Fig. 3, the second column does not have a matching column, so no submatrix can be obtained. However, the fifth column and the tenth column could construct a column cluster. When the obtained submatrix contains element 0, repeat the above steps for that submatrix until all the elements are 1.

Method $a$ differs from method $b$ in that method $a$ uses all rows as seeds once to obtain clusters. Thus method $a$ is able to obtain more biclusters. Through experimental comparison, the performance of these two methods does not differ significantly, but the operation speed of method $b$ is much lower than that of method $a$, and detailed comparison results will be given in the next section. In this thesis, Table 1 shows the step code of method $b$.

**Table 1:**
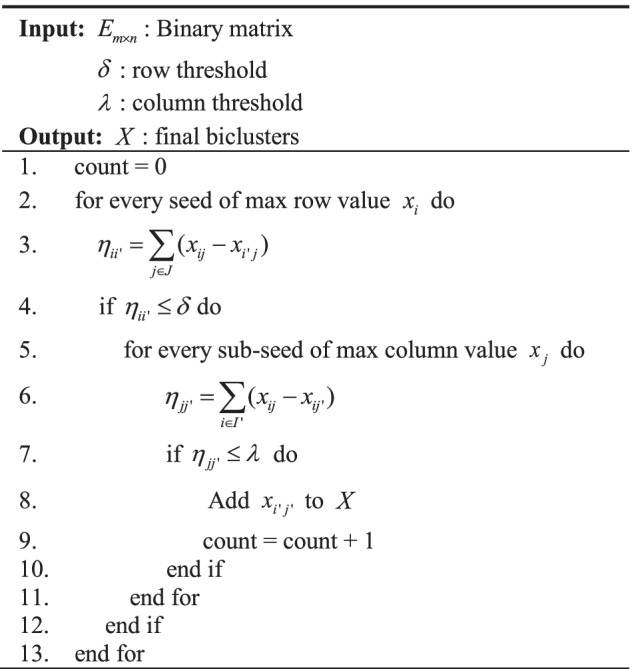
AMBB biclustering algorithm

The low time complexity is one of the characteristics of excellent biclustering algorithms. Given a matrix $M_{n \times m}$, we analyze the time complexity of the AMBB algorithm under optimal and worst cases respectively. First, the time complexity of constructing the adjacency difference matrix is $O(n(n - 1))$ ~ $O(n^{2} )$. The optimal case is when each seed only needs to be run once to get the cluster. At this time, the time complexity is $O(nm)$. There are a total of n seeds, and each seed runs m columns, of which the m is the number of data columns. Therefore, the time complexity of the AMBB algorithm is $O(\max (n^{2} ,nm))$ in optimal case. However, each seed needs to be run r/2 times in the worst case. Where r represents the number of rows in a row set. And its time complexity is $O(nm\left\lceil {r/2} \right\rceil )$. In the worst case, the time complexity of the AMBB algorithm is $O(\max (n^{2} ,nm\left\lceil {r/2} \right\rceil ))$. In other words, in the worst case, the time complexity of the algorithm may be $O(n^{2} )$. Notably, when the row threshold $\delta$ is determined to be a value, it is one run of the algorithm. When $\delta$ can be taken $t$ times, the time complexity of the algorithm is $O(t\max (n^{2} ,nm\left\lceil {r/2} \right\rceil ))$.

## Results

In this section, the performance of the AMBB algorithm is evaluated from two aspects. The first part illustrates AMBB method $a$ and $b$ from the synthetic dataset and selects the best method from the two methods to compare with the Bimax and BiBit algorithms. For synthetic dataset, the performance of the AMBB algorithm is analyzed according to the different densities of synthetic binary matrix. The second part the real datasets are used to illustrate the utility of the AMBB algorithm according to the GO enrichment analysis.

### Evaluation metric

For the binary biclustering algorithm, the commonly used evaluation metric is the match score. The details of the method are described in [9]. The Match Score is defined as follows:

3 $$S(E_{1} ,E_{2} ) = \frac{1}{{\left| {E_{1} } \right|}}\sum\limits_{{(G_{1} ,C_{1} ) \in E_{1} }} {\mathop {\max }\limits_{{(G_{2} ,C_{2} ) \in E_{2} }} \frac{{\left| {G_{1} \cap G_{2} } \right|}}{{\left| {G_{1} \cup G_{2} } \right|}}} ,$$

where $E_{1}$ and $E_{2}$ are two sets of biclusters. $E_{1}$ represents the implanted biclusters and $E_{2}$ represents the output biclusters by biclustering algorithm. $G_{1}$ is the row (gene) set of $E_{1}$. $C_{1}$ is the column (condition) set of $E_{1}$. Match score reflects the average of the maximum match scores between $E_{1}$ and $E_{2}$. When the value of $S$ is 1, this means that the biclustered $E_{1}$ is consistent with the biclustered $E_{2}$. Recovery and Relevance score are defined as $S(E_{1} ,E_{2} )$ and $S(E_{2} ,E_{1} )$, respectively.

### Synthetic dataset

Synthetic datasets are used to test the performance of AMBB. In addition, the results obtained by these two methods of AMBB are compared with the Bimax and BiBit algorithms. The synthetic dataset is divided into three sizes, namely 50*50, 100*100 and 200*200, where the density of 1’s in these three synthetic datasets ranges from 5 to 50%, increasing by 5% each time. In contrast to the experiments with synthetic datasets in [7], in this experiment, the density of 1’s is randomly distributed to simulate the uncertainty of the dataset.

The background matrix is a binary matrix, and an arbitrarily distributed value 1 indicates that the gene has important expression significance under this condition. Then we construct an all-1 matrix of size 10*10 as a bicluster. The placement rule is to allow implant in non-consecutive rows, but must be in contiguous columns. We implanted 5 biclusters in 50*50 binary background matrix and 10 biclusters in 100*100 and 200*200 binary matrix.

Since the binary synthetic datasets are all similar, we experiment with the optimal parameters set by the BiBit algorithm, $\min_{r}$ = 2 and $\min_{c}$ = 2. Through extensive experiments with the Bimax algorithm and ended up with the parameters $\min_{r}$ = 2 and $\min_{c}$ = 2 for the 50*50 and 100*100 datasets and 200*200 data sets $\min_{r}$ = 5, $\min_{c}$ = 5. Both of these algorithms are implemented in R.

**Fig. 4:**
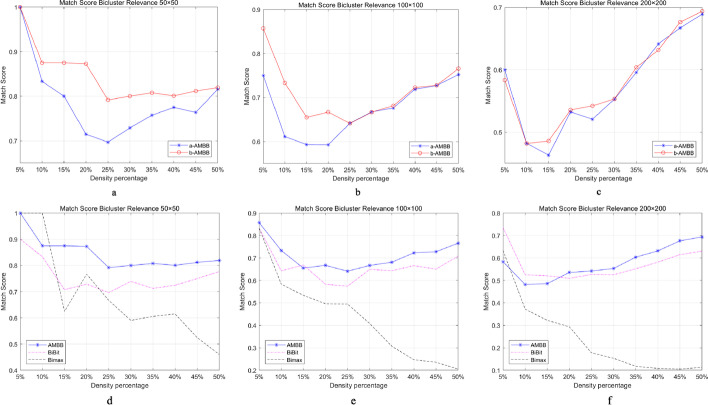
Comparison of methods for synthetic datasets. **a–c** show the comparison of two AMBB methods. **d–f** show the comparison with four biclustering algorithms

First, Fig. 4a, b and c show the performance of two ways of AMBB algorithm in synthetic dataset. Although the difference in match score is not larger, the scores of b-AMBB method are higher than a-AMBB method at low density. Furthermore, the performance of the three methods is compared. Detailed results are shown in Fig. 4d to f. For the 50*50 dataset, the results of Bimax have a poor at high density, nevertheless, the AMBB algorithm has the best performance overall. While the AMBB algorithm has a similar trend to the BiBit algorithm, the match scores of AMBB are higher than of the BiBit algorithm. In the 100*100 and 200*200 datasets, it is known that the Bimax algorithm has the lowest results, while the AMBB and BiBit performed similarly.

**Fig. 5:**
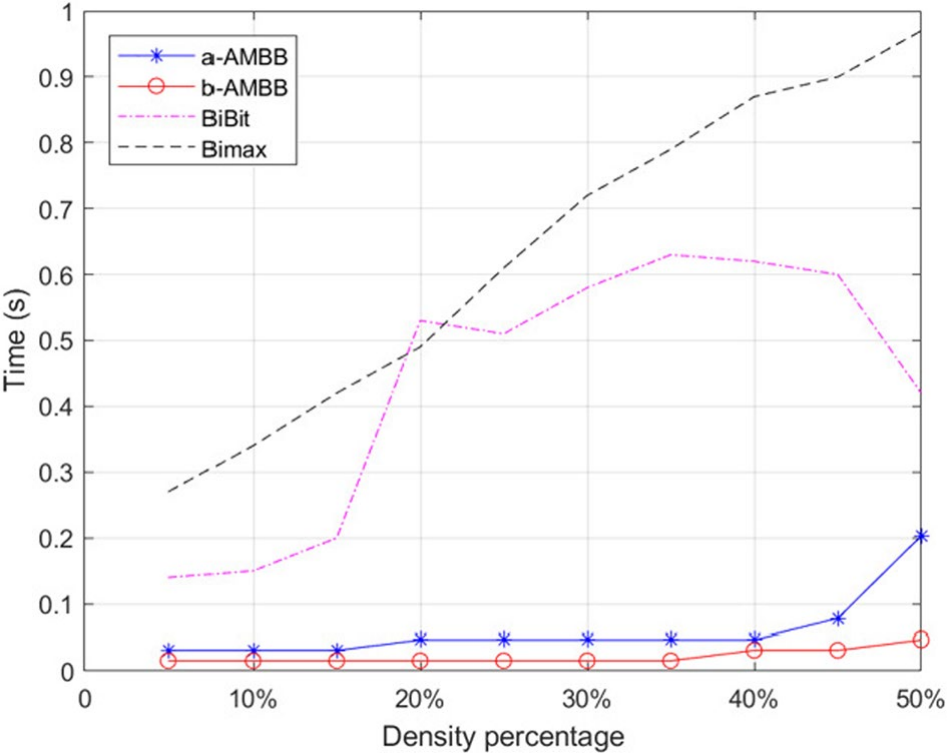
Time comparisons of the four methods are available

Figure 5 shows the running times of these four methods. The size of the synthetic dataset used is 100*100. In Fig. 5, the vertical coordinates indicate the running time of biclustering algorithms and the horizontal coordinates indicate the density of 1’s in the dataset. The two algorithms used by AMBB consume more time than the Bimax algorithm. At the same time, we can also see that the time of b-AMBB is shorter than the time of the a-AMBB algorithm. Therefore, the AMBB algorithm in this thesis mainly refers to b-AMBB method.

**Fig. 6:**
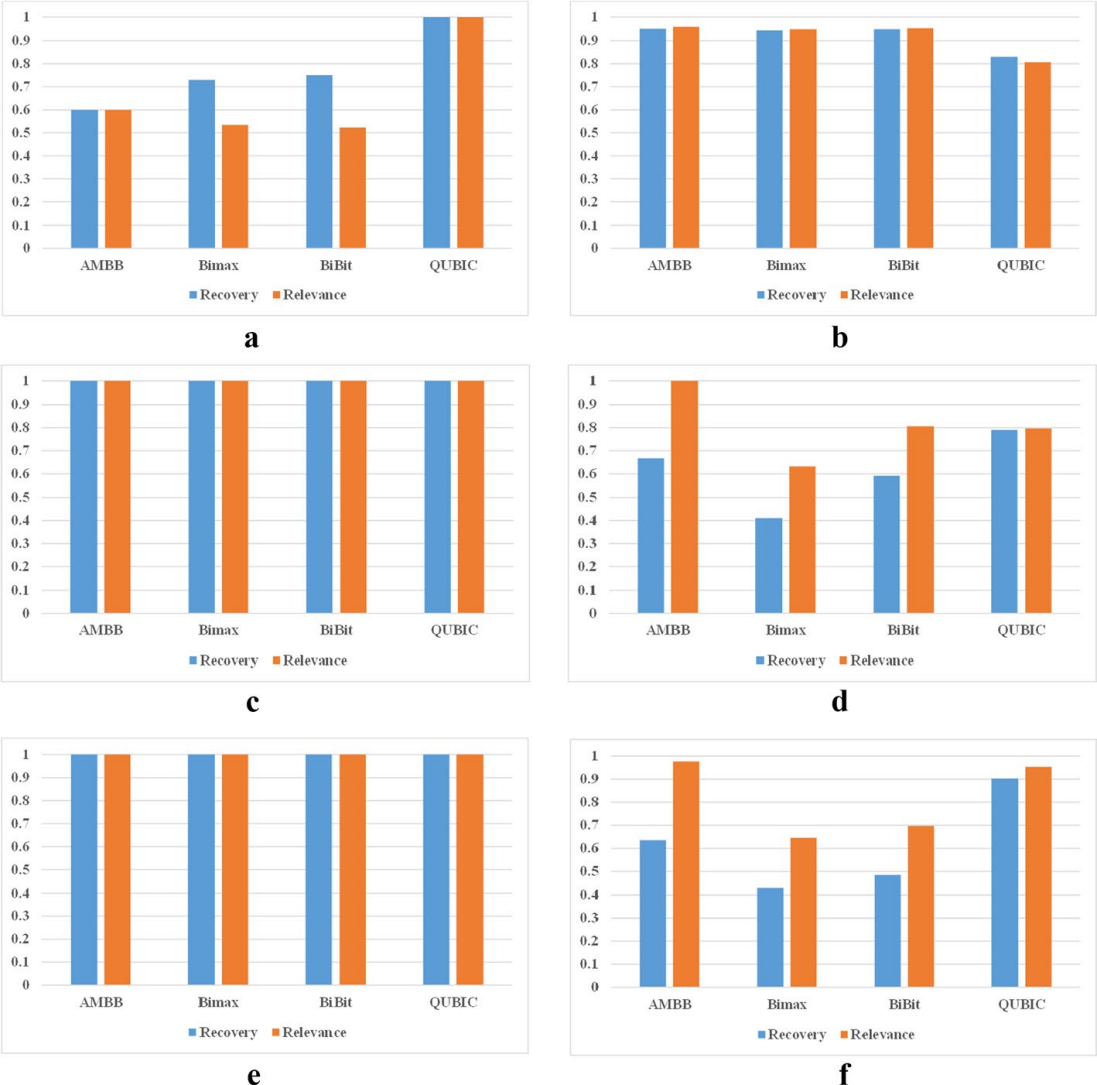
Three bicluster patterns: **a** Shift pattern with non-noise. **b** Shift pattern with noise. **c** Scale pattern with non-noise. **d** Scale pattern with noise. **e** Shift-scale pattern with non-noise. **f** Shift-scale with noise

Figure 6 shows the comparison results of the four biclustering algorithms, which are AMBB, Bimax, BiBit and QUBIC in three types of synthetic datasets. And the parameters of four biclustering algorithms are detailed in Table 2 in detail. Where m represents the minimum number of rows and columns in Bimax and BiBit algorithms and c represents the consistency level of biclusters.

**Table 2:** The value of parameters in synthetic dataset for four biclustering algorithm

Pattern	Type	AMBB	Bimax	BiBit	QUBIC
Shift-pattern	Non-noise	$\delta$ = 6	m = 5	m = 5	c = 0.55
	Noise	$\delta$ = 9	m = 5	m = 5	c = 0.55
Scale-pattern	Non-noise	$\delta$ = 4	m = 4	m = 4	c = 0.85
	Noise	$\delta$ = 13	m = 6	m = 10	c = 0.55
Shift-scale pattern	Non-noise	$\delta$ = 4	m = 4	m = 4	c = 0.85
	Noise	$\delta$ = 20	m = 7	m = 10	c = 0.45
Overlapping	Non-noise (2 × 5)	$\delta$ = 5	m = 6	m = 2	c = 0.85
	Noise (2 × 5)	$\delta$ = 9	m = 2	m = 6	c = 0.75
	Non-noise (5 × 5)	$\delta$ = 14	m = 7	m = 10	c = 0.75
	Noise (5 × 5)	$\delta$ = 19	m = 4	m = 2	c = 0.65

From Fig. 6a and b, the QUBIC algorithm is effectively at finding the implanted biclusters in a non-noise dataset, whereas the three binary biclustering algorithms are not. The major reason is that some information is lost when the data matrix is converted to a binary matrix. In dealing with noisy dataset, the score value of binary biclustering algorithm is higher than QUBIC algorithm. The reason is that the preprocessing method of binary biclustering algorithm can effectively distinguish information from noise. For the scale pattern and shift-scale pattern of biclusters shown in Fig. 6c to d, all algorithms can accurately find the implanted biclusters without noise. With the increase of noise, the scores of four biclustering algorithms are reduced. Of the four algorithms, the scores of Bimax and BiBit significantly lower. Among the biclusters obtained by the QUBIC algorithm, the amount of data belonging to the implanted biclusters is the largest among the four algorithms.. However, the biclusters obtained by the AMBB algorithm contains the least noise.

**Fig. 7:**
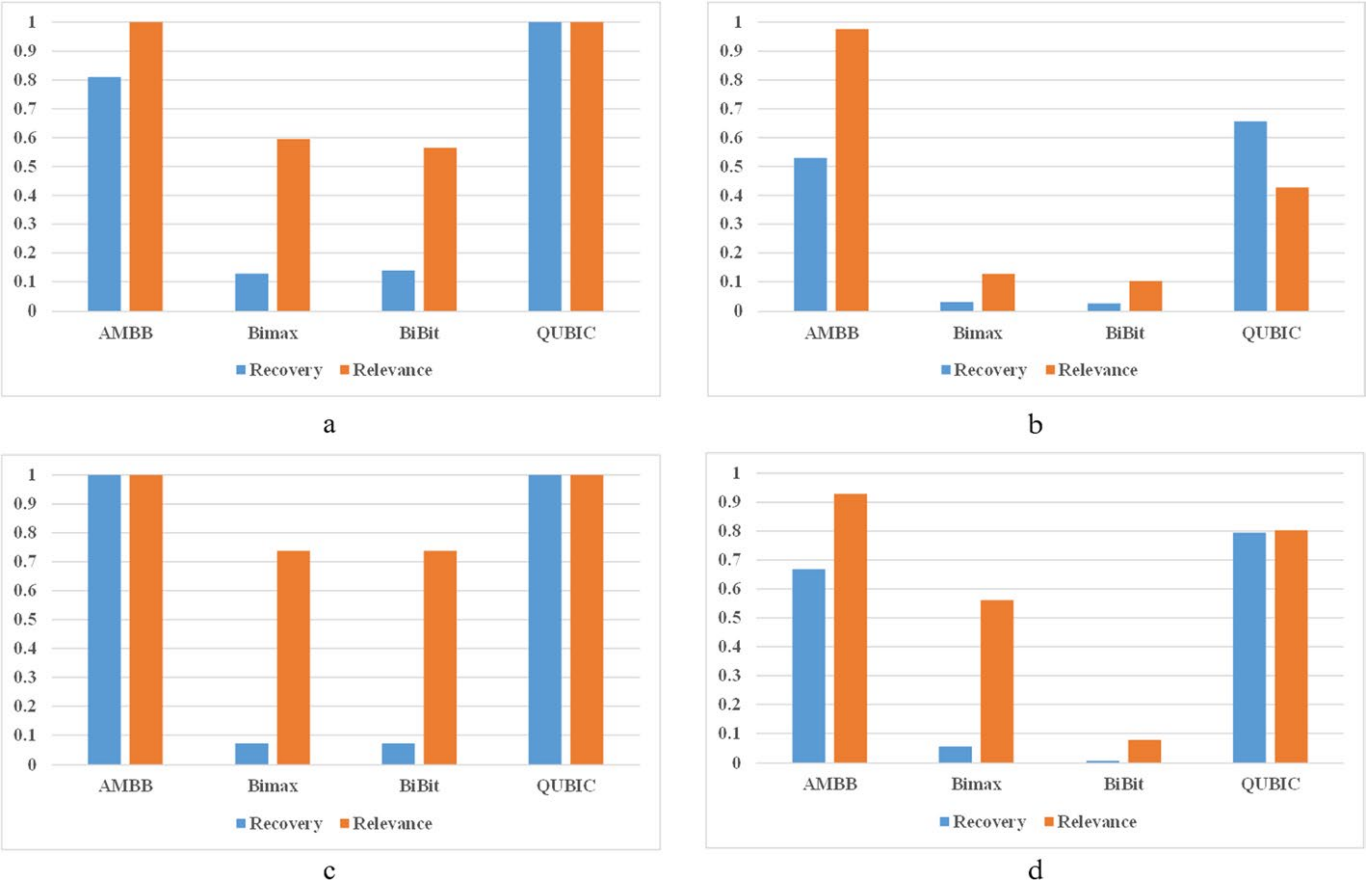
The results of overlapping bicluster experiments: **a** Non-noise data matrix with overlapping biclusters of size 2 × 5. **b** Noise data matrix with overlapping biclusters of size 2 × 5. **c** Non-noise data matrix with overlapping biclusters of size 5 × 5. **d** Noise data matrix with overlapping biclusters of size 5 × 5

We test the performance of four algorithms in overlapping biclusters. Figure 7a and b show the score values of the four algorithms in biclusters of 2*5 with overlapping sizes. The performance of the QUBIC algorithm is the best in the noise dataset, and the suboptimal method is the AMBB algorithm. In noisy datasets, the scores of the QUBIC algorithm decreased significantly. And the Relevance score of AMBB algorithm is always the highest among the four algorithms. In Fig. 7c and d, the biclusters overlapping size is 5*5. Similarly, the Relevance score of the AMBB algorithm is optimal. Compared with the QUBIC algorithm, the amount of data in the biclusters obtained by the AMBB algorithm belongs to the implanted biclusters is not much, but the noise in the obtained bicluster is very small. A part of information is lost when a data matrix is converted to a binary matrix. Therefore, the Recovery value of the AMBB algorithm is lower than the QUBIC algorithm and the Relevance value is higher than QUBIC.

By analyzing the match scores of the four biclustering algorithms in above synthetic datasets, it can be concluded that the AMBB algorithm is able to obtain the shortest running time and has the best performance compared with other algorithms.

### Real dataset

To study the utility of the AMBB algorithm, a human gene expression dataset known as Pollen [21] and a mouse gene expression dataset, namely Buettner [22] are analyzed. The homo sapiens dataset also includes GDS3715 [23] and GSE7904 [24]. And we process all datasets to remove one-mapping multiple and duplicate genes. In accordance with the preprocessing method of the Bimax algorithm [9], the dataset will first be transformed into a binary matrix before the biclustering algorithm can process it. The purpose of our analysis is to find biclusters in the dataset and to investigate the biological relevance of these genes. Table 3 exhibits the detailed information of these four datasets. To analyze real datasets, we use GO enrichment analysis [25]. This is because genes in biclustered cells are involved in biological processes. A number of methods are now available for GO enrichment analysis. We choose to use the David website because it was updated several years ago and has a lot of features and simple operations.

**Table 3:** The information of datasets

Dataset	Number of gene	Number of condition	Specie
Pollen	14,805	80	Homo
Buettner	8989	182	Mouse
GDS3715	4697	94	Homo
GSE7904	21,653	62	Homo

In this experiment, we compared four algorithms, the AMBB algorithm, QUBIC algorithm, Plaid algorithm [26] and Bimax algorithm, respectively. The QUBIC algorithm is run by the software in [27], and the Bimax algorithm is used for the package bicluster in version 4.1.2 of R. The detailed step is proposed by S. Kaiser [28]. It is worth noting that the running time of the b-AMBB method is shorter than that of the a -AMBB method. In the real data we default the AMBB algorithm to the b-AMBB method. Valid genes are used as the evaluation criteria, and genes are considered valid when the p-value is less than 0.05 [29]. The p-value is calculated as a hypergeometric distribution with the following formula:

4 $$p - value = \sum\limits_{o = n}^{N} {\frac{{\left( \begin{gathered} O \hfill \\ o \hfill \\ \end{gathered} \right)\left( \begin{gathered} T - O \hfill \\ t - o \hfill \\ \end{gathered} \right)}}{{\left( \begin{gathered} T \hfill \\ t \hfill \\ \end{gathered} \right)}}} .$$

In this formula, $O$ denotes the acquired genes and $T$ denotes the total genes in the database.

There exists a comparison result for Proportion of Genes Number. The metric is calculated as shown below:

5 $$proportion_{GN} = \frac{{R_{G} }}{{T_{G} }}.$$

where $proportion_{GN}$ represents the enrich proportion, and $R_{G}$ represents the number of identified GO items that the p-value is less than 0.05, and $T_{G}$ represents the total number of GO items. Higher values indicate more items based on the GO enrichment analysis in a bicluster and the greater the performance of the algorithm. We used David for GO enrichment analysis [30]. Table 4 shows the enrichment results of four algorithms in five datasets. The first column shows the dataset name. The second column represents biclustering algorithm, and the third column represents the enrichment ratio obtained by Eq. (5). The next column then represents the number of terms and differentially expressed terms obtained by GO enrichment analysis. And the last three columns show the p-value and items with the largest differential expression values in Biological Process (BP), Cellular Components (CC) and Molecular Function (MF). For the best results, we show them in bold. A high Ratio value does not mean that the difference expression is strong, it reflects the validity of the obtained gene cluster. In these five datasets, the ratio of the AMBB algorithm is always stable at around 0.77. Although the ratio value of the AMBB algorithm is sometimes lower than other algorithms, the p-values for terms obtained by enrichment analysis showed that the bicluster derived from the AMBB algorithm were more studing meaningful.

**Table 4:** The enrichment results of the four algorithms in the four real datasets

Dataset	Method	Ratio	Count	BP	CC	MF
				Term (*P*-value)	Term (*P*-value)	Term (P-value)
Pollen	AMBB	0.7629	621/814	**cytoplasmic translation (1.10E-65)**	**Cytoplasm (3.00E-74)**	**structural constituent of ribosome** **(3.10E-49)**
	Bimax	0.75	108/144	integrin-mediated signaling pathway (1.50E-05)	Membrane (1.00E-07)	ATP binding (1.50E-15)
	Plaid	**0.9444**	34/36	cellular lipid metabolic process (1.60E-06)	Membrane (1.30E-06)	ATPase activity, coupled to transmembrane movement of substances (3.70E-18)
	QUBIC	0.8378	124/148	acyl-CoA metabolic process (9.40E-15)	mitochondrial matrix (1.30E-07)	metalloendopeptidase activity (1.10E-18)
Buettner	AMBB	**0.7358**	518/704	**cell division (1.70E-28)**	**Nucleoplasm (6.70E-76)**	**RNA binding (2.40E-60)**
	Bimax	0.6222	28/45	Ossification (1.50E-04)	Nucleus (2.00E-04)	transmembrane transporter activity (9.50E-03)
	Plaid	0.4848	16/33	Ossification (4.90E-03)	Nucleus (1.90E-03)	nucleotidyltransferase activity (3.00E-02)
	QUBIC	0.7321	46/51	negative regulation of apoptotic process (2.70E-03)	cytoplasmic vesicle (1.50E-03)	double-stranded RNA binding (8.00E-03)
GDS3715	AMBB	0.735	294/400	**cytoplasmic translation (9.60E-21)**	**Cytosol (3.70E-28)**	**protein binding (1.00E-25)**
	Bimax	0.7769	94/121	inorganic anion transport (2.10E-08)	Membrane (3.40E-07)	ATPase activity, coupled to transmembrane movement of substances (9.10E-22)
	Plaid	0.7736	41/53	transmembrane transport (8.60E-11)	ATP-binding cassette (ABC) transporter complex (1.50E-05)	ATPase activity, coupled to transmembrane movement of substances (9.70E-16)
	QUBIC	**0.8088**	55/68	transmembrane transport (2.20E-11)	ATP-binding cassette (ABC) transporter complex (2.25E-05)	ATPase activity, coupled to transmembrane movement of substances (5.90E-17)
GSE3904	AMBB	0.8723	41/47	**structural constituent of ribosome (1.70E-38)**	**cytosolic ribosome (8.40E-46)**	structural constituent of ribosome (1.70E-38)
	Bimax	**0.9474**	54/57	transmembrane transport (2.60E-13)	intracellular membrane-bounded organelle (3.00E-04)	ATPase activity, coupled to transmembrane movement of substances (7.50E-25)
	Plaid	0.9091	30/33	transmembrane transport (8.20E-22)	integral component of membrane (1.40E-05)	ATPase activity, coupled to transmembrane movement of substances (8.70E-29)
	QUBIC	0.828	77/93	transmembrane transport (2.10E-33)	integral component of membrane (1.60E-07)	**ATPase activity, coupled to transmembrane movement of substances (1.50E-44)**

## Conclusion

In this paper, we propose a new binary biclustering algorithm for gene expression data. It uses the construction of a neighbor-joining difference matrix to obtain similar genes. This approach has better time complexity than compared algorithms, and also has high practical, which will be useful for subsequent analyses. Although the AMBB algorithm has two thresholds, the row difference threshold and the column difference threshold, at runtime for the row threshold we only give a range, and the algorithm will automatically select the optimal biclusters. For each iteration of column clustering, the column threshold is automatically increased by one until the value is $\left\lceil {r/2} \right\rceil$. After analyzing the comparison of the synthetic and real datasets, the performance of our method has been also visually demonstrated. It can be seen that the AMBB algorithm is affected by the preprocessing method from the synthetic dataset experiments. When some information is lost, the AMBB algorithm cannot obtain the optimal bicluster. Owing to the fact that the binary matrix can only show valid and non-valid genes and cannot identify the importance of valid genes. Therefore, in future studies, we will try to fuse the weights to ensure that information is not lost and cluster similar genes. Theoretically, this might produce satisfactory results.

## Data Availability

The dataset accession number by Pollen submitted at National Center for Biotechnology Information (NCBI) (http://www.ncbi.nlm.nih.gov/Traces/sra/) is SRP041736. In addition, the dataset accession number by Buettner submitted at ArrayExpresss (http://www.ebi.ac.uk/arrayexpress/) is E-MTAB-2805. Two dataset GDS3715 and GSE7904 can be found directly from NCBI.
